# The mFI-11 frailty index as a predictor of surgical outcomes in elderly patients with brain metastases

**DOI:** 10.1016/j.bas.2025.105912

**Published:** 2025-12-15

**Authors:** Stefanie Quach, Roland Coras, Florian Weissinger, Matthias Simon, Tunc Faik Ersoy

**Affiliations:** a Department of Neurosurgery, University Hospital OWL, Campus Bielefeld-Bethel, Bielefeld, Germany; b Department of Neuropathology, University Hospital Erlangen, Friedrich-Alexander-Universität Erlangen-Nürnberg, Erlangen, Germany; c Department of Hematology, Oncology and Palliative Care, University Hospital OWL, Campus Bielefeld-Bethel, Bielefeld, Germany

**Keywords:** Brain metastases, Elderly, Frailty, Comorbidities, Survival, Functional outcome

## Abstract

**Introduction:**

The vulnerability towards disease but also treatment in elderly patients has been referred to as frailty and can be measured using frailty indices, which assess functional health and comorbidities. Frailty has been shown to correlate with survival and functional outcomes in brain tumor patients.

**Research question:**

Does frailty, assessed by the 11-item modified Frailty Index (mFI-11), provide useful prognostic information in elderly patients undergoing brain metastasis surgery?

**Material and methods:**

A retrospective analysis of 139 patients aged ≥60 years who underwent brain metastasis resection (2015–2019) was conducted. Frailty was assessed using the mFI-11.

**Results:**

Increasing frailty correlated with poorer median overall survival (mFI 0: 13.8 months [95 %-CI: 8.7–18.9] vs. mFI 1–2l: 8.7 [5.8–11.6] vs. mFI≥3: 2.8 [0.9–4.6], p = 0.001) and functional outcome (postoperative KPS 80–100 %, mFI 0: 27/36 [75.0 %] vs. mFI 1–2: 44/64 [68.8 %] vs. mFI≥3: 13/38 [34.2 %], p < 0.001). Age was less prognostic. In the multivariate analyses, mFI-11 and age were not independently predictive, while KPS was. Frailty was not associated with major complications.

**Discussion:**

While frailty correlates with outcome, functional health rather than comorbidities and age is prognostic. The mFI-11's predictive ability may be largely due to its inclusion of a functional health assessment.

**Conclusion:**

Functional health (KPS) is a much stronger predictor of survival and functional outcome in the elderly than the presence of comorbidities and age, i.e. age per se and comorbidities without impact on the patient's functional health status (i.e. well-treated) should not deter from surgery. Frailty is not a predictor of complications.

## Highlights

• Frailty correlated with poorer outcomes in elderly undergoing brain metastases surgery.
• Preoperative KPS was the strongest predictor of survival and functional outcome.
• mFI was not prognostic for survival or functional outcome in multivariate analysis.
• Retrospective analysis of 139 patients ≥60 years with resected brain metastases.

## 1 Introduction

Aggressive treatment of malignant tumors in elderly patients is debatable. Therapeutic risks typically increase and survival outcomes worsen with age. However, rather than age per se the individual vulnerability towards disease but also treatment resulting from the effects of age, functional health, and comorbidities seem to underlie these observations. This vulnerability has been referred to as frailty (Doody et al., 2023) and conceptualized either as a clinical syndrome of diminished reserves and increased vulnerability to stressors (Fried et al., 2021), or as an accumulation of deficits typically quantified by comorbidities, or a combination thereof.

Frailty can be assessed using frailty indices. Some data suggest that frailty scores can offer improved predictive accuracy over traditional measures such as the Karnofsky Performance Score (KPS) and Graded-Point-Assessment (GPA) score (Kerschbaumer et al., 2022; Youngerman et al., 2018). The most commonly used Modified Frailty index (mFI) is a tool that considers both functional health status and a number of preexisting conditions such as diabetes, history of myocardial infarction, hypertension requiring medication, and previous cerebrovascular incidents with neurological deficit. The mFI-11 consists of eleven items and is based on the Accumulating Deficits Model of Frailty (Velanovich et al., 2013). The mFI is derived for each individual patient from the sum of these separate factors. A simplified 5-item score (mFI-5) is also available (Subramaniam et al., 2018).

While these scores have been developed for general geriatric or oncological populations, frailty indices have been investigated as potential predictors of surgical risks and outcomes in neurosurgical patients. Various proposed frailty measures, including the Modified Frailty Index (mFI), Clinical Frailty Scale (CFS), and Hospital Frailty Risk Score (HFRS), have been suggested to predict outcomes after brain tumor resection (Qureshi et al., 2023). Frailty indices have been investigated as survival predictors i.e. in glioblastoma patients (Cloney et al., 2016; Rahmani et al., 2020; Schneider et al., 2020; Krenzlin et al., 2021). Frailty was found to correlate with postoperative complications in patients undergoing craniotomy for brain tumors (Youngerman et al., 2018; Sastry et al., 2020). Comparing the mFI-5 to the Charlson Comorbidity Index (CCI) and the mFI-11 among brain tumor patients, the mFI-5 was found to be simple and effective in predicting postoperative mortality (Khalafallah et al., 2021). Preoperative frailty has been associated with poor survival in elderly patients with brain metastases requiring surgery (Heimann et al., 2021; Ilic et al., 2021). Although not specifically validated for this patient collective, several registry analyses suggest that in patients with metastatic brain tumors, frailty is a better predictor of postoperative outcomes than age (Dicpinigaitis et al., 2022; Colasacco et al., 2024). Outcomes are often reported either with a focus on complications and short-term postoperative mortality or as overall survival requiring longer follow-up. There is also an ongoing debate as to what defines frailty, how it should be distinguished from other factors contributing to outcome such as comorbidities and how it should be measured (Covell et al., 2024).

The current study aimed to determine if frailty as measured by mFI-11 is associated with outcome in elderly patients with brain metastases both on a short-term as well as a long-term scale, and whether its assessment improves prognostication beyond established clinical measures.

## 2 Materials and methods

### 2.1 Cohort & clinical data

One-hundred thirty-nine consecutive patients ≥60 years who underwent surgery for resection of at least one brain metastasis between January 2015 and June 2019 in our department were retrospectively analyzed for overall survival and functional outcome (i.e. postoperative KPS). Stereotactic surgeries for diagnosis confirmation were excluded. All patients were discussed in the interdisciplinary tumor board. The threshold for age was set in concordance with the perioperative GPA criteria for brain metastases (Sperduto et al., 2008, 2022). A good functional health outcome was defined as a pre- or postoperative KPS of 80–100 %, respectively. Frailty was retrospectively assessed using the 11-factor modified frailty index (Supplementary Table 1). Based on their admission mFI, patients were split into three groups: "least frail" (mFI score 0), "moderately frail" (mFI score 1–2), and "frailest" (mFI score ≥3) (Cloney et al., 2016).

Pertinent clinical data were obtained from the patients’ medical and radiology reports. Telephone interviews were – if necessary – carried out to provide complementing follow-up information. Complications were assessed using the CTCAE classification framework (Common Terminology Criteria for Adverse Events v5.0; https://ctep.cancer.gov). Complications were recorded in three categories: surgical, new persisting (≥30 days after surgery) neurological deficits, and surgery-related medical complications.

The study has been approved by the institutional review board responsible for human research and ethics committee.

### 2.2 Statistical analysis

Statistical analyses were performed with commercially available IBM SPSS Statistics for Windows software (Version 29.0, IBM Corporation, Armonk, NY, USA). Categorical parameters were analyzed using Chi-square or Fisher's exact test and we performed a ROC analysis. Kaplan-Meier-estimates were employed for survival analyses. Cox regression analysis and binary logistic regression were used for multivariate analysis. The p-value threshold for significance was set at < 0.05 for all statistical tests.

## 3 Results

### 3.1 Patient cohort

The series comprised 64 females (46.4 %) and 75 males (53.6 %). Median age was 69 years (IQR 25–75 %: 63.0–74.8). Median pre- and postoperative KPS were 80 % (IQR 25–75 %: 70–90 % and 60–90 %, respectively). The majority of the patients retained their preoperative KPS through surgery (56.5 %) and 23.2 % showed an improvement. Thirty-six (26.1 %) patients were categorized as least frail, 64 (46.4 %) as moderately frail, and 38 (27.5 %) as frailest. A more detailed description of the cohort can be found in Table 1, Table 2, and Supplementary Table 3.

**Table 1:** Univariate survival prognosis parameters.

		N	mOS (95%CI) in months	p-value
Age	≥69.0 years (median)	73 (52.3 %)	13.8 (4.5–9.4)	0.048
	<69.0 years	65 (47.7 %)	11.1 (4.9–17.3)	
Sex	Females	64 (46.4 %)	10.5 (3.8–17.1)	0.030
	Males	75 (53.6 %)	6.5 (3.4–9.5)	
Preoperative KPS^a^	80–100 %	94 (68.1 %)	11.8 (7.9–15.6)	<0.001
	≤70 %	44 (31.9)	2.7 (1.7–3.7)	
Postoperative KPS	80–100 %	84 (60.9 %)	2.4 (1.8–3.0)	<0.001
	≤70 %	54 (39.1 %)	14.6 (6.9–22.2)	
Frailty	Least frail	36 (26.1 %)	13.8 (8.7–18.9)	0.001
	Moderately frail	64 (46.4 %)	8.7 (5.8–11.6)	
	Frailest	38 (27.5 %)	2.8 (0.9–4.6)	
Histology	Lung	70 (51.0 %)	6.3 (3.2–9.5)	0.237
	Breast	17 (12.3 %)	8.7 (2.0–15.5)	
	Other	51 (61.3 %)	11.7 (6.4–16.9)	
Primary known	Yes	42 (32.7 %)	6.0 (2.2–9.9)	0.997
	No	95 (67.3 %)	8.7 (5.2–12.2)	
Multiple brain metastases	Yes	77 (55.8 %)	5.3 (2.1–8.5)	0.112
	No	61 (44.2 %)	11.6 (7.4–15.8)	
Extracerebral metastases^b^	Yes	84 (45.4 %)	7.8 (3.5–12.1)	0.842
	No	44 (54.6 %)	8.1 (4.1–12.1)	
Perioperative GPA^c^	1	68 (11.4 %)	4.7 (1.6–7.8)	0.161
	2	42 (46.0 %)	11.1 (4.3–17.9)	
	3	18 (24.7 %)	13.6 (8.1–19.0)	
	4	0		
Postoperative radiotherapy	Yes	97 (73.8 %)	11.8 (8.6–15.0)	<0.001
	No	41 (26.2 %)	1.6 (1.1–2.1)	
Postoperative systemic therapy^d^	Yes	68 (55.0 %)	14.6 (7.1–22.1)	<0.001
	No	67 (45.0 %)	3.2 (2.0–4.4)	
Major surgical complication	Yes	13 (6.2 %)	2.5 (0.0–8.5)	0.079
	No	125 (93.8 %)	8.6 (5.5–11.7)	
Major neurological complication	Yes	20 (5.3 %)	2.5 (0.0–11.8)	0.041
	No	118 (94.7 %)	8.7 (5.1–12.4)	
Major medical complication	Yes	14 (7.2 %)	1.5 (0.8–2.3)	<0.001
	No	124 (92.8 %)	9.7 (6.7–12.7)	

a KPS – Karnofsky Performance Status.

b Information missing for 10 patients.

c GPA – Graded Prognostic Assessment, information missing for 10 patients.

d Information missing for 2 patients.

**Table 2:** Univariate prognosis parameters for functional outcome.

		Discharge KPS 80–100 %	p-value
Age	≥69.0 years (median)	36/73 (49.3 %)	0.003
	<69.0 years	48/65 (74.0 %)	
Sex	Females	45/64 (70.3 %)	0.035
	Males	39/74 (52.7 %)	
Preoperative KPS^a^	80–100 %	82/94 (87.2 %)	<0.001
	≤70 %	2/44 (4.5 %)	
Frailty	Least frail	27/36 (75.0 %)	<0.001
	Moderately frail	44/64 (68.8 %)	
	Frailest	13/38 (34.2 %)	
Histology	Lung	44/70 (62.9 %)	0.754
	Breast	11/17 (64.7 %)	
	Other	29/51 (56.9 %)	
Primary known	Yes	27/42 (64.3 %)	0.555
	No	56/95 (58.9 %)	
Extracerebral metastases^b^	Yes	50/84 (59.5 %)	0.233
	No	31/44 (70.5 %)	
Multiple brain metastases	Yes	41/74 (55.4 %)	0.039
	No	43/61 (70.5 %)	
Perioperative GPA^c^	1	32/68 (47.1 %)	<0.001
	2	34/42 (81.0 %)	
	3	15/18 (83.3 %)	
	4	0	
Major surgical complication	Yes	3/13 (2.3 %)	0.003
	No	81/125 (64.8 %)	
Major neurological complication	Yes	5/20 (25.0 %)	<0.001
	No	79/118 (70.0 %)	
Major medical complication	Yes	3/14 (21.4 %)	0.001
	No	81/124 (65.3 %)	

a KPS – Karnofsky Performance Status.

b Information missing for 10 patients.

c GPA – Graded Prognostic Assessment, information missing for 10 patients.

### 3.2 Short-term outcome: functional outcome

**Fig. 1:**
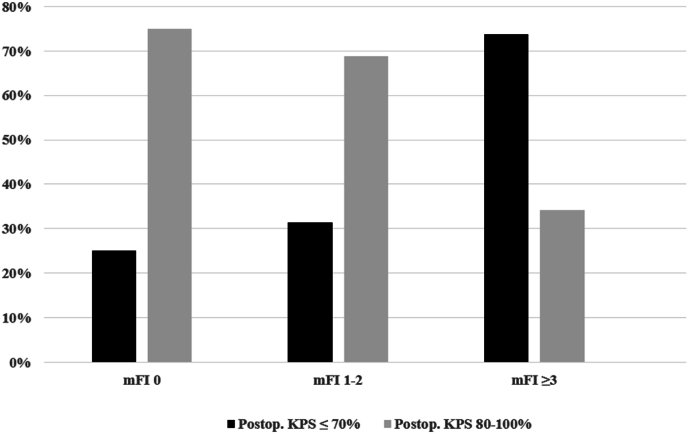
Increasing frailty correlates with a poorer postoperative functional outcome (i.e. KPS ≤70 %, p < 0.001).

Increasing frailty was strongly associated with a poorer functional outcome (postoperative KPS 80–100 %, least frail: 27/36 [75.0 %] vs. moderately frail: 44/64 [68.8 %] vs. frailest: 13/38 [34.2 %], p < 0.001, Fig. 1). Likewise, a poorer presurgical functional health status (i.e. KPS ≤70 %) was a very strong prognostic parameter (postoperative KPS 80–100 %, preoperative KPS ≤70 %: 2/44 [4.5 %] vs. preoperative KPS 80–100 %: 82/94 [87.2 %], p < 0.001). The ROC analysis showed that preoperative KPS predicted postoperative functional outcome better than mFI (area under the curve AUC KPS: 0.877 [95 %-CI: 0.807–0.947] vs. AUC mFI: 0.670 [95 %-CI: 0.576–0.764], Fig. 4). Other predictors for a better functional outcome included a younger age (i.e. < 69.0 years), single brain metastasis, a higher perioperative GPA score and avoidance of major complications (Table 2).

Frailty was not a predictor of major complications: surgical complication (least frail: 2/36 [5.6 %] vs. moderate frail 5/65 [7.7 %] vs. severe frail 6/38 [15.9 %], p = 0.268); new persisting neurological deficit (least frail: 4/36 [11.1 %] vs. 7/64 [10.9 %] vs. 9/38 [23.7 %], p = 0.167); and medical complication (least frail: 2/36 [5.6 %] vs. 5/64 [7.8 %] vs. 7/38 [18.4 %], p = 0.131).

### 3.3 Long-term outcome: overall survival

Median overall survival (mOS) was 7.8 (95 %-CI: 5.2–10.5) months. At the time of the analysis, 112 patients had already died. Cause of death was available for 83 patients (60.1 %); 41 patients (29.7 %) had died as a consequence of their CNS-disease.

**Fig. 2:**
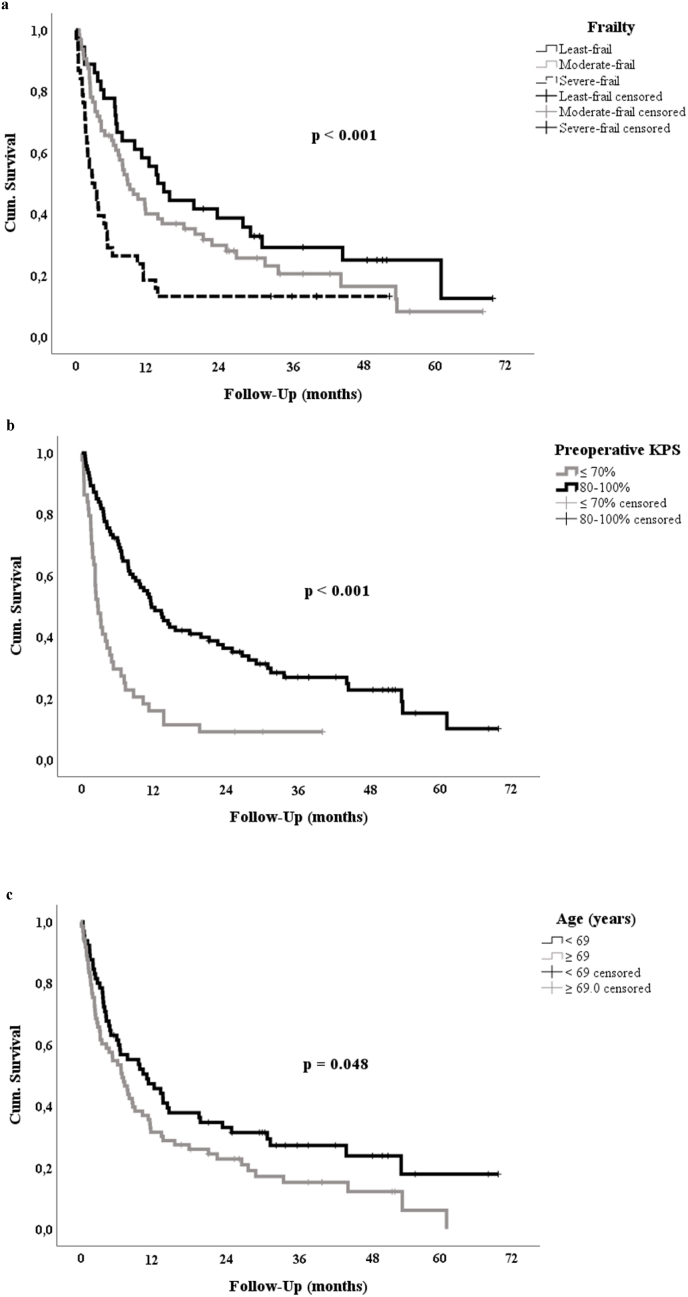
Kaplan-Meier estimates for preoperative predictors of overall survival.

Increasing frailty was strongly correlated with poorer survival (mOS least frail: 13.8 [95 %-CI: 8.7–18.9] months vs. moderately frail: 8.7 [5.8–11.6] vs. frailest: 2.8 [0.9–4.6], p = 0.001). Impaired preoperative functional health (i.e. KPS ≤70 %) was also strongly prognostic (mOS KPS ≤70 %: 2.7 [95 %-CI: 1.7–3.7] months vs. KPS 80–100 %: 11.8 [7.9–15.6], p < 0.001). Other prominent prognostic factors included age, gender, postoperative KPS, postoperative radiotherapy (RT) and systemic treatment (ST) as well as new persisting major neurological deficits and medical complications (Table 1 and Fig. 2).

### 3.4 Multivariate analysis: comparison of clinical measures

**Fig. 3:**
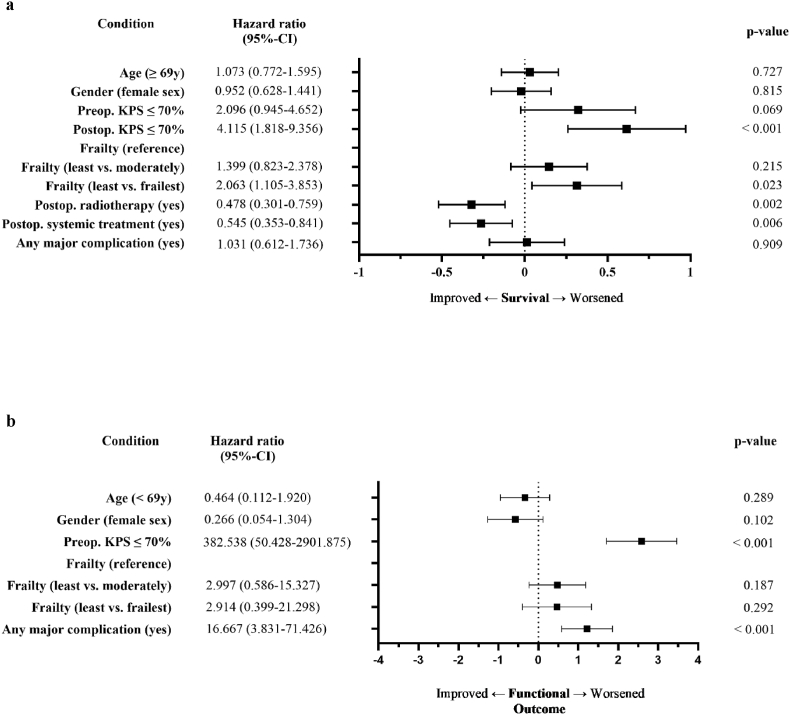
Multivariate analysis: Comparison of clinical measures.

Multivariate cox regression analysis of all parameters correlating significantly with survival in the univariate analysis (for statistical purposes, major complications were assessed together as “any major complication”) yielded postoperative KPS and postoperative treatment as well as occurrence of any major complication as independent prognostic factors. KPS proved to be the strongest survival predictor. Binary logistic regression analysis of all parameters significantly linked to functional outcome in the univariate analysis identified preoperative KPS as well as any major complication as independent prognosticators. The mFI-11 score was not independently predictive in any of the multivariate analyses (Fig. 3).

**Fig. 4:**
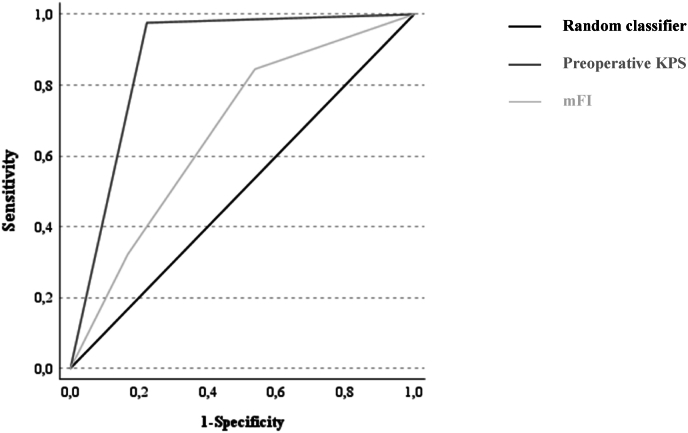
ROC analysis of preoperative KPS and mFI as predictors of functional outcome.

## 4 Discussion

This study investigates surgical outcomes in elderly patients undergoing brain metastases resection. We retrospectively analyzed data from 139 patients, assessing frailty using the modified Frailty Index (mFI) and correlating it with overall survival and functional outcome based on the Karnofsky Performance Score (KPS).

Overall survival of elderly patients remains grim (7.8 months). However, approximately 40 % of the patients in our cohort survived at least one year, with a quarter of them living longer than two years. Our results indicate that preoperative functional health (i.e. the KPS) rather than frailty (or age) is the strongest prognostic factor for survival and functional outcome.

Frailty has been investigated before as an outcome predictor in patients with brain metastases. In a series of 180 geriatric patients with surgically treated brain metastases, Heimann et al. found frailty to impact overall survival, while comorbidities did not reach statistical significance and functional status was not analyzed separately (Heimann et al., 2021). In a follow-up study of 141 patients with NSCLC metastases, the same group identified sarcopenia, frailty, KPS, age, and multiple BM as significant and independent predictors of poor OS. Of note, KPS showed the highest hazard ratio of 2.5 (Ilic et al., 2021).

In our study, both frailty and preoperative functional health status correlated with survival during an adequately long follow-up. The present analysis expands on previous results, showing that frailty and preoperative functional status also influence postoperative functional health. Functional health is linked to quality of life and therefore a therapeutic goal in its own right (Martin et al., 2012). In the context of malignant disease, it is a prerequisite for further therapy required for prolonged survival as shown by the present study (Table 1; mOS without postoperative treatment ≤3 months). Of note, extent of disease did not appear to influence outcome in this cohort, which implies that, rather than the presence of systemic metastases, the availability of further oncological treatment options should be considered as a selection criterion for surgery. The retrospective design of the study, however, does not enable us to evaluate the role of genetic driver mutations or immune-/targeted-therapies for particular disease subtypes (Supplementary Table 3). In large registry studies frailty has been shown to correlate with all types of postoperative complications (Youngerman et al., 2018; Dicpinigaitis et al., 2022; Skandalakis et al., 2023; Telera et al., 2023). Interestingly, in our cohort, no strong influence of frailty on complications could be identified, although inclusion criteria defining major complications according to the CTCAE framework or the Clavien-Dindo classification are comparable (Dindo et al., 2004). While sample size may be the cause of this discrepancy, the impact of frailty on the risk of incurring complications does not appear to be significant enough to generally exclude frail patients from having surgery. Nevertheless, major complications entail the risk of relevant functional deterioration, which could prevent future therapy and jeopardize survival (Table 1; mOS <3 months). Therefore, utmost care should be taken to prevent complications in particular in frail patients.

Investigating the role of frailty in oncological disease has been hampered by a lack of standardization of frailty assessments in part resulting from discrepant concepts of frailty. Ultimately, frailty as a clinical syndrome overlaps with age, functional status and comorbidities. Some indices try to address single aspects of this syndrome: The Clinical Frailty Scale was developed as a clinical scoring system for geriatric patients (Rockwood et al., 2005). It is a subjective assessment that correlates strongly with KPS (Kerschbaumer et al., 2022). Similar to KPS, dependence on others for activities of daily living is an important cut-off (Supplementary Table 2). The strong analogies to KPS evaluation led us to rely on the more routinely used mFI scale. On the other side of the spectrum, the Hospital Frailty Risk Score tries to quantify frailty using all patient-specific ICD-10 codes and weighing them for relevance (Hannah et al., 2020). While it can predict short-term outcomes such as complications, length of stay, hospital charges, non-routine discharge, and readmission (Jimenez et al., 2023; Kazemi et al., 2024), it is not a frailty index per se, as acute symptoms are taken into account although they are not part of a decreased physiological reserve (Covell et al., 2024). The Risk Analysis Index (RAI) has a large functional component focusing on cognition, mobility and assistance in activities of daily living in addition to life-threatening comorbidities (Hall et al., 2017). It can be used as a clinical questionnaire or retrospectively from diagnostic code datasets (Dicpinigaitis et al., 2024) and predicts 30-day mortality after resection of brain metastases (Skandalakis et al., 2023). It does however require a thorough documentation of i.e. unintentional weight loss, poor appetite or shortness of breath. The decision to operate or not requires an easily applicable evaluation tool, ideally using information routinely acquired in a clinical setting.

We found it intriguing that the KPS proved to be a stronger outcome predictor than the mFI-11 score and age. From a statistical point of view, the KPS and not the mFI-11 score or age was identified as an independent predictor of both, functional and survival outcomes. Since the mFI includes a list of comorbidities as well as a functional health status assessment similar to the KPS, this may imply a limited predictive role for comorbidities. In other words, age and comorbidities alone should not deter from recommending surgery as long as the latter are well-treated and compensated. This is an important conclusion and message for everyday neurosurgery practice.

Our study is limited by the size of the cohort and a pre-selection bias since only patients deemed “operable” were analyzed. Its strengths include a follow-up with an adequate event number and evaluating not only overall survival, but also functional health. We feel that out study addresses a clinically relevant question, i.e. whether elderly, often multimorbid patients should have their intracranial metastasis resected. Our results appear to show that this decision should be prominently based on the patient's functional health status rather than age alone, frailty as assessed with the mFI-11 score, and the mere presence of comorbidities.

## 5 Conclusions

mFI-11 is a valid tool for predicting survival and functional outcomes in elderly patients with brain metastases undergoing surgery. Frailty was not a predictor of major complications. Our results also demonstrate, however, that these findings may largely reflect the impact of the patient's functional health status (which is included in the assessment of mFI) and not the role of comorbidities or age. Age and comorbidities without impact on a patient's functional health status should not necessarily deter from surgery in the elderly.

## Consent to participate

The responsible institutional research committee and local law do not require informed consent for this study.

## Consent to publish

No personal data is published in this manuscript; not applicable for this study.

## Author contributions

Conceptualization: [Tunc F. Ersoy, Matthias Simon]; Methodology: [Tunc F. Ersoy, Matthias Simon], Formal analysis and investigation: [Tunc F. Ersoy, Stefanie Quach]; Writing - original draft preparation: [Tunc F. Ersoy, Stefanie Quach]; Writing - review and editing: [Tunc F. Ersoy, Stefanie Quach, Florian Weissinger, Matthias Simon]; Resources: [Roland Coras]; Supervision: [Tunc F. Ersoy].

All authors read and approved the final manuscript.

## Ethics approval

*This study was performed in line with the principles of the Declaration of Helsinki. Approval was* granted *by the Ethics Committee of* Ärztekammer Westfalen-Lippe und der Westfälischen Wilhelms-Universität Münster, Germany *(*Az, 2021-073-f-S*).*

## Funding

The authors declare that no funds, grants, or other support were received during the preparation of this manuscript.

## Declaration of competing interest

The authors declare that they have no known competing financial interests or personal relationships that could have appeared to influence the work reported in this paper.

## Data Availability

The datasets generated during and/or analyzed during the current study are available from the corresponding author on reasonable request.
