# Supplementary material for: The mFI-11 frailty index as a predictor of surgical outcomes in elderly patients with brain metastases
Source: Brain Spine. 2025 Dec 15;6:105912. doi: 10.1016/j.bas.2025.105912 (PMC12771347; doi:10.1016/j.bas.2025.105912)
Supplement: Multimedia component 1 [file mmc1.docx]

**Supplementary**

| **Table 1:** 11-Item Modified Frailty Index | |
| --- | --- |
| **Item** | **Index weight** |
| Functional health (dependent) | 1 |
| Diabetes mellitus | 1 |
| Chronic obstructuve pulmonary disease or pneumonia | 1 |
| Congestive cardiac failure | 1 |
| Myocardial infarction | 1 |
| Previous percutaneous procedure or angina pectoris | 1 |
| Arterial hypertension | 1 |
| Peripheral vascular disease | 1 |
| Impaired sensorium | 1 |
| Transient ischemic attack or cerebrovascular accident | 1 |
| Neurological deficit after previous cerebrovascular accident | 1 |

**Table 2:** Clinical Frailty Scale

| 1 | Very fit — robust, active, energetic, well motivated and fit; these people commonly exercise regularly and are in the most fit group for their age |
| --- | --- |
| 2 | Well — without active disease, but less fit than people in category 1 |
| 3 | Well, with treated comorbid disease — disease symptoms are well controlled compared with those in category 4 |
| 4 | Apparently vulnerable — although not frankly dependent, these people commonly complain of being “slowed up” or have disease symptoms |
| 5 | Mildly frail — with limited dependence on others for instrumental activities of daily living |
| 6 | Moderately frail — help is needed with both instrumental and non-instrumental activities of daily living |
| 7 | Severely frail — completely dependent on others for the activities of daily living, or terminally ill |

**Table 3:** Histological subtypes and molecular characteristics

| **Lung (69)** | *Adenocarcinoma (29)* |
| --- | --- |
|  | EGFR: 4 wt, 25 unknown |
|  | BRAF: 1 Mut, 3 wt, 25 unknown |
|  | KRAS: 1 Mut, 1 wt, 27 unknown |
|  | ALK/ROS1: 4 wt, 25 unknown |
|  | MET: 2 wt, 27 unknown |
|  | RET: 3 wt, 26 unknown |
|  | NTRK: 2 wt, 27 unknown |
|  | PD-L1: 1 negative, 26 unknown |
|  | *Squamous cell ca. (3)* |
|  | EGFR: 1 wt, 2 unknown |
|  | ALK/ROS: 1 wt, 2 unknown |
|  | PD-L1: 1 negative, 2 unknown |
|  | *Large cell/neuroendocrine ca. (2)* |
|  | *Pleomorphous ca. (1)* |
|  | *Adenosquamous ca. (1)* |
|  | *Small cell ca. (12)* |
|  | *Unknown subtype (20)* |
| **Breast (19)** | ER: 7 +, 11 -, 1 unknown |
|  | PR: 5 +, 13 -, 1 unknown |
|  | HER2: 13 +, 5 -, 1 unknown |
| **Gastrointestinal (17)** | *Adenocarcinoma (17)* |
|  | Colon (10) |
|  | RAS: 1 Mut, 1 wt, 8 unknown |
|  | Esophagus (3) |
|  | Pharynx (2) |
|  | Pancreas (1) |
|  | Stomach (1) |
|  | *Squamous cell ca. (1; esophagus)* |
| **Melanoma (8)** | BRAF: 1 Mut, 2 wt, 5 unknown |
| **Renal (7)** |  |
| **Ovary/cervix/endometrial cancer (6)** |  |
| **Urothelial (4)** |  |
| **Unknown primary (4)** |  |
| **Prostate (2)** |  |
| **Thyroid (1)** |  |
| **Sarcoma (1)** |  |

**ca., carcinoma**
